# Draft genome of the leopard gecko, *Eublepharis macularius*

**DOI:** 10.1186/s13742-016-0151-4

**Published:** 2016-10-26

**Authors:** Zijun Xiong, Fang Li, Qiye Li, Long Zhou, Tony Gamble, Jiao Zheng, Ling Kui, Cai Li, Shengbin Li, Huanming Yang, Guojie Zhang

**Affiliations:** 1 College of Forensic Medicine, Xi’an Jiaotong University, Xi’an, Shaanxi 710061 China; 2 China National GeneBank, BGI–Shenzhen, Shenzhen, 518083 China; 3 State Key Laboratory of Genetic Resources and Evolution, Kunming Institute of Zoology, Chinese Academy of Sciences (CAS), Kunming, Yunnan 650223 China; 4 Centre for GeoGenetics, Natural History Museum of Denmark, University of Copenhagen, Øster Voldgade 5-7, 1350 Copenhagen, Denmark; 5 Department of Biological Sciences, Marquette University, Milwaukee, WI 53201 USA; 6 Centre for Social Evolution, Department of Biology, University of Copenhagen, Universitetsparken 15, DK-2100 Copenhagen, Denmark; 7 BGI-Shenzhen, Shenzhen, 518083 China; 8 James D. Watson Institute of Genome Sciences, Hangzhou, 310058 China

**Keywords:** Gekkota, Leopard gecko, *Eublepharis macularius*, Genome sequencing, Assembly

## Abstract

**Background:**

Geckos are among the most species-rich reptile groups and the sister clade to all other lizards and snakes. Geckos possess a suite of distinctive characteristics, including adhesive digits, nocturnal activity, hard, calcareous eggshells, and a lack of eyelids. However, one gecko clade, the Eublepharidae, appears to be the exception to most of these ‘rules’ and lacks adhesive toe pads, has eyelids, and lays eggs with soft, leathery eggshells. These differences make eublepharids an important component of any investigation into the underlying genomic innovations contributing to the distinctive phenotypes in ‘typical’ geckos.

**Findings:**

We report high-depth genome sequencing, assembly, and annotation for a male leopard gecko, *Eublepharis macularius* (Eublepharidae). Illumina sequence data were generated from seven insert libraries (ranging from 170 to 20 kb), representing a raw sequencing depth of 136X from 303 Gb of data, reduced to 84X and 187 Gb after filtering. The assembled genome of 2.02 Gb was close to the 2.23 Gb estimated by k-mer analysis. Scaffold and contig N50 sizes of 664 and 20 kb, respectively, were comparable to the previously published *Gekko japonicus* genome. Repetitive elements accounted for 42 % of the genome. Gene annotation yielded 24,755 protein-coding genes, of which 93 % were functionally annotated. CEGMA and BUSCO assessment showed that our assembly captured 91 % (225 of 248) of the core eukaryotic genes, and 76 % of vertebrate universal single-copy orthologs.

**Conclusions:**

Assembly of the leopard gecko genome provides a valuable resource for future comparative genomic studies of geckos and other squamate reptiles.

**Electronic supplementary material:**

The online version of this article (doi:10.1186/s13742-016-0151-4) contains supplementary material, which is available to authorized users.

## Data description

### Sample collection and sequencing

**Fig. 1:**
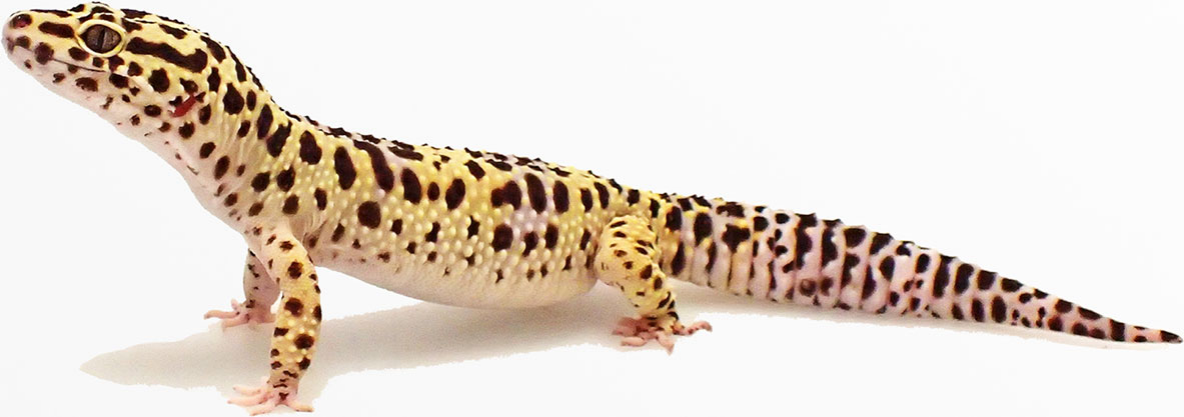
Example of a Leopard gecko *Eublepharis macularius* (image from Tony Gamble)

Genomic DNA was extracted from the tail tissue of a male leopard gecko (*Eublepharis macularius*: NCBI taxonomy ID 481883; specimen ID TG1477) (Fig. 1). All tissues were collected in accordance with University of Minnesota animal use protocols 0810A50001 and 1108A03545. This animal was captive born from 30+ generations of inbreeding of a strain originating from animals of Indian origin at the Woodland Park Zoo (Seattle) and imports from Pakistan at the National Zoo (Washington, DC) [1]. A total of seven paired-end libraries with a gradient insert size ranging from 170 to 20 kb were constructed and sequenced on an Illumina HiSeq 2000 platform according to the manufacturer’s instructions (Illumina, San Diego, California, USA). For long insert size libraries (2, 5, 10 and 20 kb), the sequenced read length was 49 bp, while for short insert size libraries (170, 500 and 800 bp), the sequenced read lengths were 100 and 150 bp (Table 1). A total of 303 Gb (136X) raw sequences were eventually obtained (Table 1). Before assembly, strict quality control was performed for raw reads using SOAPfilter, a software application in the SOAPdenovo package [2], which included removing low-quality reads and duplicate reads arising from PCR amplification during library construction. Sequencing errors were corrected using the k-mer frequency method in SOAPec (version 2.02) [2]. After filtering and correction, 187 Gb (84X) high-quality sequences were obtained for genome assembly (Table 1).

**Table 1:** Summary statistics of leopard gecko sequence data derived from paired-end sequencing of seven insert libraries using an Illumina HiSeq 2000 platform

Library insert size (bp)	# Lane	Read length (bp)	Raw data		High-quality data	
			Total bases (Gb)	Sequencing depth (X)	Total bases (Gb)	Sequencing depth (X)
170	2	100	60.25	27.03	57.20	25.66
500	2	150	76.08	34.13	59.36	26.63
800	1	150	27.84	12.49	15.90	7.13
2000	3	49	58.04	26.04	34.88	15.65
5000	2	49	33.96	15.24	10.99	4.93
10,000	2	49	29.17	13.09	5.09	2.28
20,000	1	49	17.33	7.78	4.07	1.83
Total	13		302.66	135.78	187.49	84.11

Note: Sequencing depth was calculated based on a genome size of 2.23 Gb. High-quality data were obtained by filtering raw data for low-quality and duplicate reads and correcting sequencing errors

### Genome assembly

We first performed a 17-mer analysis [2] to estimate the leopard gecko genome size using 54 Gb clean sequences from 170 and 500 bp insert size libraries. Briefly, reads were divided into sliding short sequences of 17 bp, overlapping by 16 bp, with the exception of the first base pair. The count distribution of 17-mers followed a Poisson distribution (Additional file 1). The genome size was estimated as 2.23 Gb for *E. macularius* by dividing the total number of 17-mers by the peak of distribution (Table 2).

**Table 2:** Statistics of genome size estimation by 17-mer analysis. The genome size was estimated according to the formula: Genome size = # Kmers/Peak of depth

Genome	Kmer length (bp)	# Kmers	Peak of depth	Estimated genome size (bp)	Data used (bp)
*Eublepharis macularius*	17	46,813,180,882	21	2,229,199,089	53,806,135,250

We then assembled a high-quality leopard gecko genome using SOAPdenovo (version 2.0) [2] in three steps: contig construction, scaffolding, and gap filling. In the contig construction step, SOAPdenovo was used to a de Bruijn graph by dividing high-quality reads from short insert libraries into kmers in which paired-end information was ignored, and kmers were then merged, tips clipped, bubbles merged, and low coverage links removed. Next, contigs displaying unambiguous connections in de Bruijn graphs were collected. A series of kmer lengths were tested and a 33-mer was selected to generate a contig assembly with the longest N50 value. In the scaffolding step, reads from both small and large insert libraries were mapped to contig sequences to construct scaffolds using distance information from read pairs, with the requirement that at least three read pairs were used to form a reliable connection between two contigs. To close intra-scaffold gaps (the gap filling step), overlapping paired-end reads from the 170 bp insert library were first connected using COPE [3], then Kgf [2] was employed to close gaps using these connected reads together with reads from other short insert size libraries. An additional local assembly for reads with one end of a read pair uniquely aligned to a contig and the other end located within the gap was performed using GapCloser [2]. The end result was a leopard gecko genome assembly with a total length of 2.0 Gb and scaffold and contig N50s of 664 and 20 kb, respectively, which is comparable to the previously reported *Gekko japonicus* genome assembly (Table 3) [4]. Comparison of assembly N50s for the leopard gecko genome with eleven previously published reptile genomes (*Anolis carolinensis* [5], *Python molurus bivittatus* [6], *Ophiophagus hannah* [7], *Alligator sinensis* [8, 9], *Alligator mississippiensis*, *Gavialis gangeticus*, *Crocodylus porosus* [10], *Chelonia mydas*, *Pelodiscus sinensis* [11], *Pogona vitticeps* [12], and *Chrysemys picta bellii* [13]) further confirmed that our results were of comparable or better quality (Table 4).

**Table 3:** Comparison of genome features between *Eublepharis macularius* and *Gekko japonicus*

Genome features	*Eublepharis macularius*	*Gekko japonicus*
Assembled genome size (Gb)	2.02	2.55
Scaffold N50 (kb)	664	685
Contig N50 (kb)	20.0	21.1
Gene Number	24,755	22,487
Repeat content (% of genome)	42.18	48.94

**Table 4:** Summary statistics of key parameters for 13 reptile genomes

Species	Common name	Sequencing technology	Sequence coverage	Assembly size (Gb)	Contig N50 (kb)	Scaffold N50 (kb)	References
*Anolis carolinensis*	Green anole lizard	Sanger	6.0X	1.78	79.9	4033	[5]
*Alligator sinensis*	Chinese alligator	NGS	109.0X	2.30	23.4	2188	[8]
*Chrysemys picta bellii*	Western painted turtle	Sanger + NGS	18.0X	2.59	11.9	5212	[13]
*Chelonia mydas*	Green sea turtle	NGS	82.3X	2.24	20.4	3778	[11]
*Pelodiscus sinensis*	Soft-shell turtle	NGS	105.6X	2.21	21.9	3331	[11]
*Python molurus bivittatus*	Burmese python	NGS	20.0X	1.44	10.7	208	[6]
*Ophiophagus hannah*	King cobra	NGS	28.0X	1.66	4.0	226	[7]
*Alligator mississippiensis*	American alligator	NGS	156.0X	2.17	7.0	509	[10]
*Gavialis gangeticus*	Indian gharial	NGS	81.0X	2.88	14.2	127	[10]
*Crocodylus porosus*	Saltwater crocodile	NGS	74.0X	2.12	32.8	205	[10]
*Gekko japonicus*	Japanese gecko	NGS	131.3X	2.55	21.1	685	[4]
*Pogona vitticeps*	Australian dragon lizard	NGS	179.1X	1.82	31.3	2290	[12]
*Eublepharis macularius*	Leopard gecko	NGS	135.8X	2.02	20.0	664	

### Estimation of genome completeness

We evaluated the completeness of the assembly using CEGMA [14] and BUSCO [15], which quantitatively assess genome completeness using evolutionarily informed expectations of gene content. CEGMA assessment showed that our assembly captured 225 (91 %) of the 248 ultra-conserved core eukaryotic genes, of which 210 (85 %) were complete. BUSCO analysis showed that 58 and 18 % of the 3023 expected vertebrata genes were identified as complete and fragmented, respectively, while 24 % were considered missing in the assembly. Both assessment methods showed that our assembly was more complete than the previously reported *Gekko japonicus* genome assembly (Tables 5 and 6).

**Table 5:** Coverage of core eukaryotic genes (CEGs) in the gecko genome assessed by CEGMA. All CEGs were divided into four groups based on their degree of protein sequence conservation. Group 1 contains the least conserved CEGs and group 4 contains the most conserved

	*Eublepharis macularius*		*Gekko japonicus*	
	Proteins	Completeness (%)	Proteins	Completeness (%)
Complete	210	84.68	182	73.39
Group 1	53	80.30	51	77.27
Group 2	49	87.50	44	78.57
Group 3	52	85.25	43	70.49
Group 4	56	86.15	44	67.69
Partial	225	90.73	202	81.45
Group 1	59	89.39	58	87.88
Group 2	52	92.86	47	83.93
Group 3	55	90.16	48	78.69
Group 4	59	90.77	49	75.38

**Table 6:** Summarized benchmarks in the BUSCO assessment

	*Eublepharis macularius*		*Gekko japonicus*	
BUSCO benchmark	Number	Percentage	Number	Percentage
Total BUSCO groups searched	3023		3023	
Complete single-copy BUSCOs	1746	57.757	1528	50.546
Complete duplicated BUSCOs	31	1.025	27	0.893
Fragmented BUSCOs	551	18.227	580	19.186
Missing BUSCOs	726	24.016	915	30.268

### Repeat annotation

We combined a homology-based and *de novo* method to identify transposable elements (TEs) and other repetitive elements in the leopard gecko genome. Using the homology-based method, we identified known TEs using RepeatMasker [16] to search against the Repbase TE library (RepBase21.01) [17] and RepeatProteinMask within the RepeatMasker package to search against the TE protein database. In the *de novo* method, we first constructed a *de novo* leopard gecko repeat library using RepeatModeler (http://www.repeatmasker.org/RepeatModeler.html, version 1.0.5) and Piler [18], and the de novo TE library was subsequently used by RepeatMasker to annotate repeats in the leopard gecko genome. Finally, we used TRF [19] to predict tandem repeats, with the following parameters: Match = 2, Mismatch = 7, Delta = 7, PM = 80, PI = 10, Minscore = 50. Overall, we identified a total of 851 Mb of non-redundant, repetitive sequences, accounting for 42 % of the leopard gecko genome. The most predominant elements were long interspersed nuclear elements (LINEs), which accounted for 30 % of all TE sequences and 13 % of the genome (Table 7).

**Table 7:** Summary statistics of annotated repeats in the leopard gecko genome assembly

Repeat type	Total repeat length (bp)	Percentage of genome
DNA	69,961,035	3.47
LINE	255,603,529	12.67
SINE	106,528,475	5.28
LTR	64,149,381	3.18
Unknown	390,378,296	19.35
Total	850,708,938	42.18

### Gene prediction

We combined homology-based, *de novo*, and transcriptome-based methods to predict protein-coding genes in the leopard gecko genome.

In the homology-based methods, we downloaded the gene sets of *Taeniopygia guttata*, *Homo sapiens*, *Anolis carolinensis*, *Pelodiscus sinensis* and *Xenopus tropicalis* from the Ensembl database (release-73). We first aligned these homologous protein sequences to the leopard gecko genome assembly using TBLASTN with an E-value cutoff of 1e-5, and linked the BLAST hits into candidate gene loci with GenBlastA [20]. We then extracted genomic sequences of candidate loci, together with 3 kb flanking sequences, using GeneWise [21] to determine gene models. Finally, we filtered pseudogenes that had only one exon with frame errors, as these loci were probably derived from retrotransposition.

In the *de novo* method, we randomly selected 1000 leopard gecko genes with intact open reading frames (ORFs) and the highest GeneWise score from the homology-based gene set to train the Augustus [22] gene prediction tool with default parameters. Augustus was then used to perform a *de novo* gene prediction on repeat-masked genome sequences. Gene models with incomplete ORFs and small genes with a protein-coding length <150 bp were filtered out. Finally, a BLASTP search of predicted genes was performed against the SwissProt database [23]. Genes with matches to SwissProt proteins containing any one of the following keywords were filtered: transpose, transposon, retro-transposon, retrovirus, retrotransposon, reverse transcriptase, transposase, and retroviral.

Transcriptome-based gene prediction was then performed using leopard gecko RNA-seq data from liver, salivary gland, scent gland, and skin tissues obtained from the NCBI database (accession number SRR629643, ERR216315, ERR216316, ERR216322, ERR216325, ERR216304 and ERR216306) [24]. Tophat (v1.3.3) was used to align the RNA-seq reads against the leopard gecko genome assembly to identify splice junctions, and cufflinks (v2.2.1) was used to assemble transcripts using the aligned RNA-seq reads [25].

Finally, the results of homology-, *de novo-*, and transcriptome-based analyses were merged to yield a non-redundant reference gene set based on a priority order of transcriptome-based evidence > homology-based evidence > *de novo*-based evidence. We employed an in-house annotation pipeline to merge the gene data as follows:

1. A Markov model was estimated with 1000 high-quality genes, which were previously used to train Augustus, using the trainGlimmerHMM tool included in the GlimmerHMM software package [26]. The coding potential of each transcript assembled from the transcriptome data was then identified using the Markov model. Transcripts with complete ORFs were extracted and multiple isoforms from the same locus were collapsed by retaining the longest ORF.
2. These non-redundant ORFs were then integrated with homology-based gene models to form the core gene set using a custom script. If a gene model with a higher priority overlapped with a model with a lower priority (overlapping length >100 bp), the latter was removed. If two gene models with the same priority overlapped, the one with a longer ORF was preferred.
3. Homology-based gene models not supported by transcriptome-based evidence but supported by homologous evidence from at least two species were added to the core gene set.
4. *De novo*-based gene models not supported by homology-based and transcriptome-based evidence were added to the core gene set where significant hits (BLASTP E-value <1e-5) for non-transposon proteins in the SwissProt database were obtained.

As a result of these steps, a total of 24,755 non-redundant protein-coding genes were annotated in the leopard gecko genome assembly.

### Functional annotation of protein-coding genes

We assigned names to 93.59 % of all leopard gecko protein-coding genes by searching against the function databases TrEMBL and SwissProt [23] using BLASTP (Table 8). We then searched the leopard gecko protein sequences against the Kyoto Encyclopaedia of Genes and Genomes (KEGG) database [27] using BLASTP to identify molecular pathways that the genes might be involved in. Protein domains and motifs were annotated using InterProScan (version 5.16) [28] using seven different models (Profilescan, blastprodom, HmmSmart, HmmPanther, HmmPfam, FPrintScan and PatternScan). This revealed that 20,958 of the predicted leopard gecko proteins had conserved functional motifs. We also obtained 1028 Gene Ontology (GO) [29] terms that were assigned to 15,873 leopard gecko proteins from the corresponding InterPro entry.

**Table 8:** Statistics for functional annotation

Functional database	Number of genes annotated
InterPro	20,958 (84.66 %)
GO	15,873 (64.12 %)
KEGG	16,172 (65.33 %)
TrEMBL	23,139 (93.47 %)
SwissProt	22,347 (90.27 %)

### Availability and requirements

- Project name: Leopard gecko genome annotation scripts
- Project home page: https://github.com/gigascience/paper-xiong2016
- Operating systems: Linux
- Programming language: PERL
- Other requirements: none
- License: MIT
- Any restrictions to use by non-academics: none

## Additional file

Additional file 1: Frequency distribution of 17-mer analysis. 17-mers are counted from a subset of paired-end reads from 170 bp and 500 bp libraries. The peak depth is 21X. The total number of 17-mers present in this subset is 46,813,180,882. The genome size, estimated by dividing the total number of 17-mer by the peak depth, is 2.229 Gb. (PDF 179 kb)
