# Supplementary figures and images for: Draft genome of the leopard gecko, Eublepharis macularius
Source: Gigascience. 2016 Oct 26;5:47. doi: 10.1186/s13742-016-0151-4 (PMC5080775; doi:10.1186/s13742-016-0151-4)

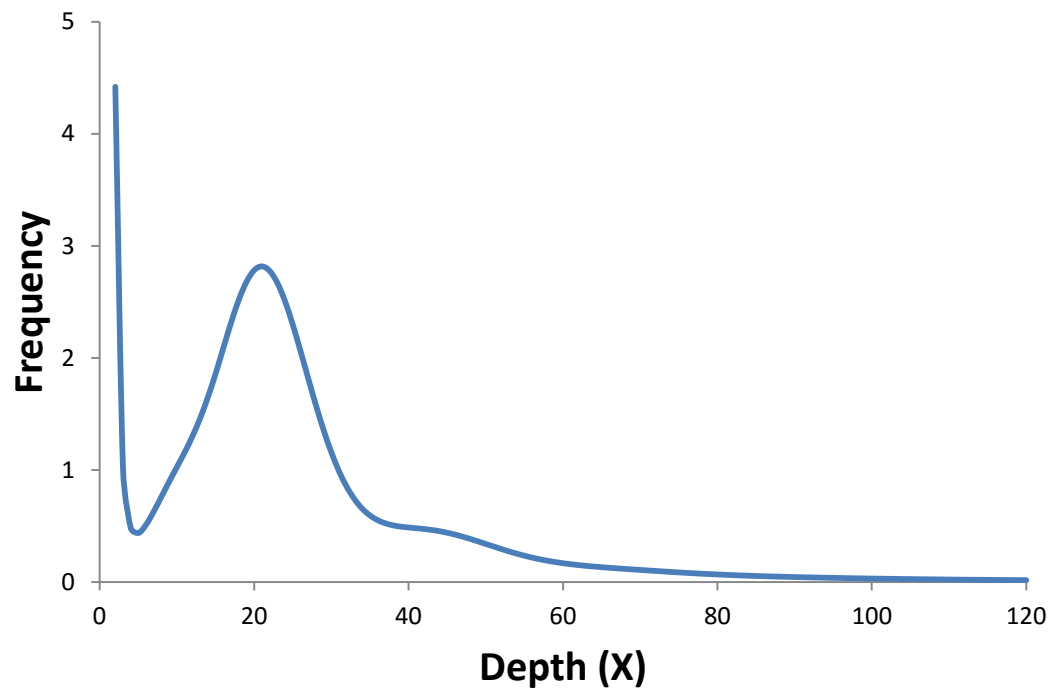

Supplement: Additional file 1: — Frequency distribution of 17-mer analysis. 17-mers are counted from a subset of paired-end reads from 170 bp and 500 bp libraries. The peak depth is 21X. The total number of 17-mers present in this subset is 46,813,180,882. The genome size, estimated by dividing the total number of 17-mer by the peak depth, is 2.229 Gb. (PDF 179 kb) [file 13742_2016_151_MOESM1_ESM.pdf]
